# Lightweight Aggregate Made from Dredged Material in Green Roof Construction for Stormwater Management

**DOI:** 10.3390/ma9080611

**Published:** 2016-07-23

**Authors:** Rui Liu, Reid Coffman

**Affiliations:** College of Architecture and Environmental Design, Kent State University, Kent, OH 44242, USA; rcoffma4@kent.edu

**Keywords:** lightweight aggregate, dredged material, stormwater management

## Abstract

More than 1.15 million cubic meters (1.5 million cubic yards) of sediment require annual removal from harbors and ports along Ohio’s Lake Erie coast. Disposing of these materials into landfills depletes land resources, while open water placement of these materials deteriorates water quality. There are more than 14,000 acres of revitalizing brownfields in Cleveland, U.S., many containing up to 90% impervious surface, which does not allow “infiltration” based stormwater practices required by contemporary site-based stormwater regulation. This study investigates the potential of sintering the dredged material from the Harbor of Cleveland in Lake Erie to produce lightweight aggregate (LWA), and apply the LWA to green roof construction. Chemical and thermal analyses revealed the sintered material can serve for LWA production when preheated at 550 °C and sintered at a higher temperature. Through dewatering, drying, sieving, pellet making, preheating, and sintering with varying temperatures (900–1100 °C), LWAs with porous microstructures are produced with specific gravities ranging from 1.46 to 1.74, and water absorption capacities ranging from 11% to 23%. The water absorption capacity of the aggregate decreases as sintering temperature increases. The LWA was incorporated into the growing media of a green roof plot, which has higher water retention capacity than the conventional green roof system.

## 1. Introduction

**Figure 1:**
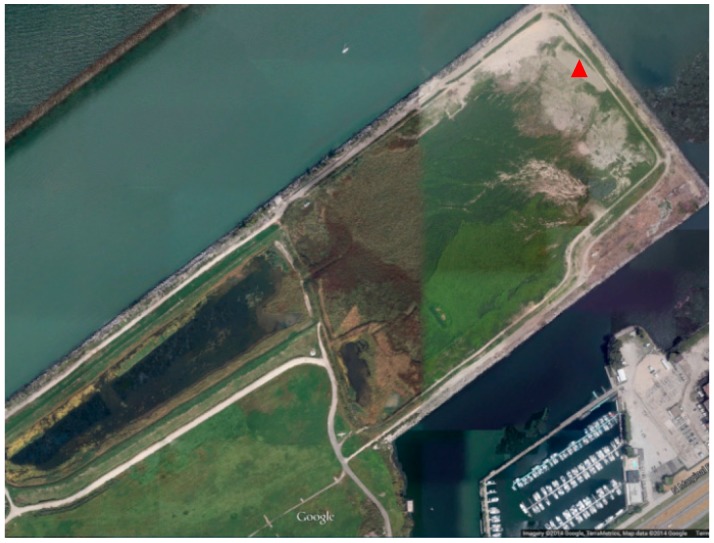
Confined disposal facility (CDF) for dredged material in Cleveland (Imagery @2015 Google, TerraMetrics, Map data @2015).

Sedimentation build-up in rivers, lakes, and oceans impairs movement of aquatic vehicles through navigational channels. These large ships and barges require a critical depth to be able to navigate successfully through large bodies of water. As a result, rivers, lakes and oceans need to be dredged periodically. There are hundreds of harbors and ports built along the Great Lakes and other inland lakes across the country. Annually, millions of cubic meters of dredged material are removed from these harbors and ports to maintain economic viability and public safety. For example, each year, more than 1.15 million cubic meters (1.5 million cubic yards (CY)) of sediment requires removal from fifteen federal harbors and numerous smaller ports built along Ohio’s Lake Erie coast. Disposal of the dredged material into landfills is costly and depletes valuable land, while open water placement (occurring in most harbors in Ohio) has the potential to deteriorate water quality through siltation, increased turbidity, and mobilization of potential contaminants. The economic and beneficial uses of dredged material in the built environment have already attracted public interest. In Cleveland, dredged material is disposed of in a 104-acre confined disposal facility (CDF) (Figure 1) maintained by the Cleveland-Cuyahoga County Port Authority. The Ohio EPA is monitoring the levels of heavy metal and organic contaminants in the facility. However, additional capacity of the facility is needed to accommodate the 225,000 CY of annual sediment disposal in order to keep the Port of Cleveland operational and economically viable.

In Cleveland, there are more than 14,000 acres of brownfields, many with over 90% impervious surface. Impervious surface is commonly employed on post-industry lands to abate soil pollution. However, this practice conflicts with “infiltration”, the principle required by contemporary site-based stormwater strategies. In addition, research shows that devaluation and destabilization of neighborhoods are around these unremediated brownfields, and the impervious surface increases flooding concerns in combined sewer overflow areas where many brownfields are located. Green infrastructure (GI)—such as green roof, bioswales, and rain gardens—emphasizing hydrological retention and evapotranspiration through soil and plant water interactions could provide a flexible and affordable solution to remediate urban brownfield stormwater and pollutant management.

Two forms of dredged material, raw and sintered, could potentially be reused in the built environment. Raw dredge is sediment in its original unaltered form, and sintered dredge is the manufacturing and processing of the sediment into an industrial “baked” commercial product. The application of raw dredge is most common and occurs within several categories of beneficial use, including (1) habitat; (2) beach nourishment; (3) parks and recreation; (4) agriculture forestry, horticulture and aquaculture; (5) strip-mine reclamation/solid waste management; and (6) construction/industrial development [1,2,3,4,5,6,7]. The beneficial use of raw dredge material stems from the growing demand for construction materials and dwindling inland sources. A common practice in many countries around the world is to use dredged material as (1) concrete aggregates (sand and gravel); (2) backfill material or material for producing bituminous mixtures and mortar (sand); (3) raw material for brick manufacturing (clay with less than 30% sand); (4) ceramics, such as tile (clay) pellets for insulation or lightweight backfill or aggregate (clay); (5) raw material for the production of riprap or blocks for the protection of dikes and slopes against erosion (rock, mixture); and (6) raw material for the production of compressed blocks for security walls at military installations and for gated communities and home subdivisions [3,4,5].

Lightweight aggregate (LWA) can potentially be made from sintering dredged material, which can create an ecologically beneficial product as well as an economical alternative compared to the presently produced LWA. Bremmer and Ries [8] (p. 1) stated “lightweight concrete [aggregate] is an effective material in enhancing sustainability as compared to other construction materials. In addition LWAs can provide important environmental components for sustainability when it is used for improving water quality as well as when it is used for the growing medium for green roof use to combat the urban heat island effect.” Currently, LWAs are usually made of shale, clay, slate, or even sludge from water or wastewater treatment plants [9]. 

LWA is frequently used in horticulture [10] and green infrastructure practices [11]. Green roofs take advantage of the lightweight and hydrological retention capacities of expanded clay, slate, and other materials to control stormwater runoff [12,13,14]. Extensive vegetative roofs are characterized by their shallow growing depth and their low maintenance needs [15]. The growing media depth of an extensive roof averages less than 15 cm (6 inches). Being this shallow, extensive roofs use LWA to optimize stormwater management solutions for new and retrofit roofs, while reducing the need for permanent irrigation systems. The plants fitting an extensive vegetative roof environment are shallow rooted sedums, herbs, and grasses. Intensive vegetative roofs are characterized by their greater growing depth and improved rooting zone capacity created by the physical and structural properties of LWA. The growing medium depth of an intensive roof averages 15 cm (>6 inches). This extra depth also allows for greater plant and wildlife biodiversity. Intensive vegetative roofs can grow increased varieties of plants, from mosses and short sedums to shrubs and trees. Vegetated roof systems that use LWA in the growing course provide soil structure and water retentive capacity while being lighter in weight than other soil products. This trade practice is extremely common in the global, North American, and Great Lakes markets. The most common LWA is made from sintering freshly mined clay and shale. Dry climates, which are also usually urban environments, tend to incorporate LWA in the extensive roofs. When applied to buildings, green roofs aid in urban pollution abatement while supplying a range of site-based ecological services, including energy, biodiversity, and nutrient cycling [14,15,16,17]. When used in bioswales and rain gardens, LWA improves water retention, pollution removal, and plant success. The dredged material may supply raw mineral materials for aggregate production, which has high hydrological retention capacity for GI construction.

This study developed a lab testing protocol to explore the beneficial use potential of dredged material for stormwater management on urban brownfields. Specifically, two objectives were addressed in the study: (1) evaluating the suitability of dredged material from Cleveland Harbor for use in green infrastructure (e.g., green roof, rain garden) in brownfield remediation; and (2) preparing and producing “green” LWA with high porous surface and inner microstructure, which is made from dredged material. The “green” LWA was produced through preheating and sintering based on parameters determined from thermal and chemical analysis. The potential for using this LWA in green roof construction was evaluated in the lab and through field study. This project addresses the issues caused by the disposal of dredged material in Lake Erie by investigating the reuse of the material in GI for stormwater management and brownfield remediation. The practice of GI in brownfield is expected to address stormwater pollution and reduce combined sewer overflow events and the cost of wastewater treatment in Cleveland.

## 2. Materials and Experimental Design

**Figure 2:**
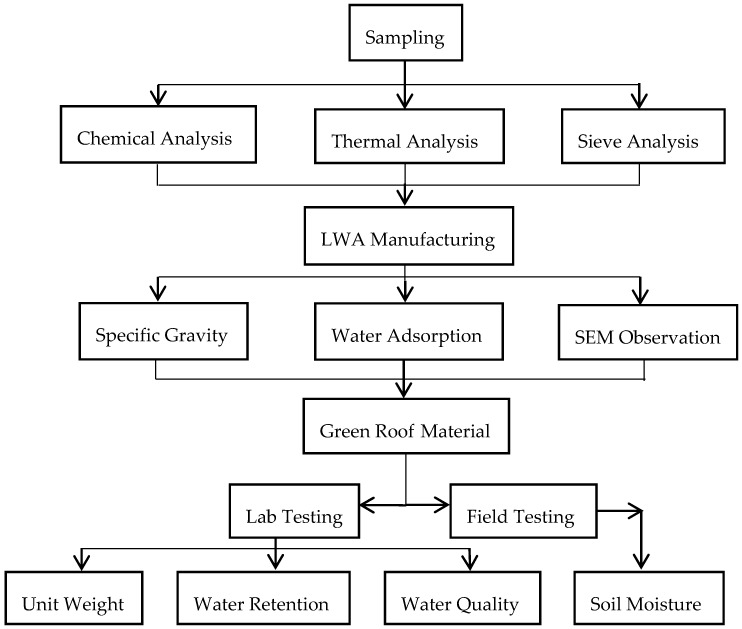
Experimental design.

Dredged material was sampled from a CDF managed by the Harbor of Cleveland to evaluate if the dredged material was suitable for LWA manufacturing and green infrastructure construction. The dredged material samples were dried and pulverized. Small pellets were made in the lab. After dried in the air, the pellets were sintered in a furnace with varying temperatures between 550 °C and 1150 °C. After the completion of the sintering, the pellets were cooled down to room temperature and tested for its physical properties, i.e., specific gravity and water absorption capacity. Microstructure of the samples was observed using a scanning electronic microscope (SEM). The samples with the best performances were used to replace the LWA in a commercial green roof material, Rooflite^®^. The unit weight, water retention capacity, and drained water quality were tested in the lab. Two green roof microcosms, one constructed using LWA made from dredged material, and the other with Rooflite^®^, were implemented in the field. The soil moistures of the two green roof microcosms were monitored for six weeks. The experimental design is illustrated in Figure 2.

### 2.1. Sampling

**Figure 3:**
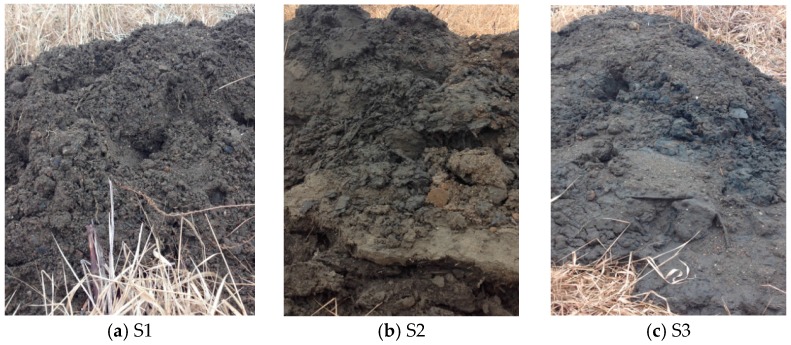
Dredged material samples.

A total of four samples were taken from the CDF. Three of them (S1, S2, and S3, shown in Figure 3) were sampled in January 2015 from the same location at a depth of 0–0.91 m (0–3 feet), 0.9–1.83 m (3–6 feet), and 1.83–2.74 m (6–9 feet), respectively, indicated as a red triangle in Figure 1. The fourth sample (S4) was taken in August 2015, which was newly dredged material poured at the CDF in June 2015. Samples of S1, S2, and S3 are shown in Figure 3. S1 is very sandy; S2 and S3 have relatively high silt and clay contents, as does S4.

### 2.2. Heavy Metals and Sieve Analysis

Aluminum, antimony, arsenic, barium, beryllium, cadmium, calcium, chromium, cobalt, copper, iron, lead, magnesium, nickel-soluble salts, potassium, selenium, silver, sodium, thallium, vanadium, and zinc were measured using EPA Method SW846 6010B. Mercury was tested according to EPA Method SW846 7471A. Total cyanide and chromium were evaluated using EPA Method 335.2 and EPA Method SW846 7196A, respectively. The contents of gravel, coarse sand, fine sand, silt, and clay were determined by sieve analysis. 

### 2.3. Thermal Analysis 

Only S3 was taken for thermal analyses and elemental scanning because it was sampled at a depth of 1.83–2.74 m (6–9 feet) and many of its properties were not disturbed by the natural environment. The thermal analyses included thermogravimetric analysis (TGA) and differential scanning calorimetry (DSC). TGA was used to measure the weight loss of the dredged material sample as a function of increasing temperature. The sample was heated from 20 to 705 °C at 10 °C per minute. The temperature was held for one minute at 705 °C. The organic contents were determined from the TGA results. The DSC is a thermoanalytical technique which measures the endothermic reaction of a sample as a function of temperature. The DSC curve shows evaporation of adsorbed water and crystal water as the temperature increases. In the DSC testing, the temperature was held at 20 °C for 1 min, then the sample was heated from 20 °C to 600 °C at 10 °C per min. Multi-element scan can determine the concentration of up to 81 elements in a sample preparation, providing a means to identify the elemental composition of the dredged material sample.

### 2.4. LWA Production

**Figure 4:**
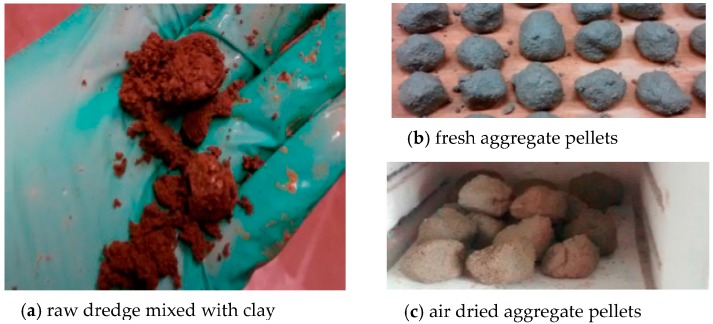
Aggregates manufacturing.

**Figure 5:**
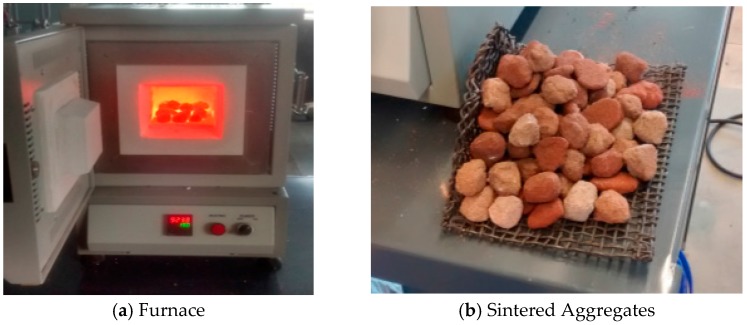
Sintering of aggregates.

The dredged material samples were air-dried and pulverized. The LWA was produced by mixing the dredged material with water, and firing in a furnace at different temperatures. S1, S2, and S3 were mixed with water and 0%, 5%, 10%, 15%, and 20% (by weight) clay (Figure 4a). The clay acted as a bonding agent, especially for sandy sample material taken from the top layer in the disposal facility. The water content was adjusted to achieve a desired plasticity with cohesive properties. Due to the high clay content in S4, no additional clay was used in the mixture. The raw dredge mixtures were used to make small pellets with 10 mm (0.5 in.) diameter or less (Figure 4b) in the lab. The fresh pellets were air-dried for minimum 24 h before firing. The dried pellets were packed in a furnace (Sentro Tech ST 1150-458 high temperature box furnace, Strongsville, OR, USA) with a chamber sizing 102 mm × 127 mm × 203 mm (4" × 5" × 8") and fired according to a schedule determined by the thermal analysis. The samples were sintered at varying temperatures ranging from 550 to 1150 °C (Figure 5).

The aggregate manufacturing process included an initial screening to remove unusable materials, forming pellets by grinding, mixing with water and other mineral admixtures as needed, extruding, and firing in a kiln or furnace. The aggregates can be crushed and graded to suit the needs of customers.

### 2.5. Specific Gravity and Water Absorption Rate

Specific gravity is the ratio of the density of the aggregates made from dredged material to the density of water (Equation (1)). Dry weight (DW), saturated weight (SW), and submerged weight (SmW) of the sintered aggregates can be measured. According to the Archimedes’ principle, specific gravity of the sintered aggregates can be determined using Equation (2). The water absorption capacity (AC) can be measured using Equation (3).

(1) $$\text{SG}=\frac{\rho_{\text{sample}}}{\rho_{w\text{ater}}}$$

where SG = specific gravity, $\rho_{\text{sample}}=\text{density of samples}$, and$\;\rho_{\text{sample}}=\text{density of water}$ (62.5 lbs/ft^3^, 1 kg/m^3^ at 4 °C).

(2) $$\text{SG}=\frac{\text{DW}}{\text{SW}-\text{SmW}}$$

where SG = specific gravity of sintered aggregates, SW = saturated weight of sintered aggregates, SmW = submerged weight of sintered aggregates, and DW = dry weight of sintered aggregates.

(3) $$\text{AC}=\frac{\text{SW}-\text{DW}}{\text{DW}}\times 100\%$$

where AC = water absorption capacity, SW = saturated weight of sintered aggregates, SmW=submerged weight of sintered aggregates, and DW = dry weight of sintered aggregates.

### 2.6. Scanning Electronic Microscope (SEM)

**Figure 6:**
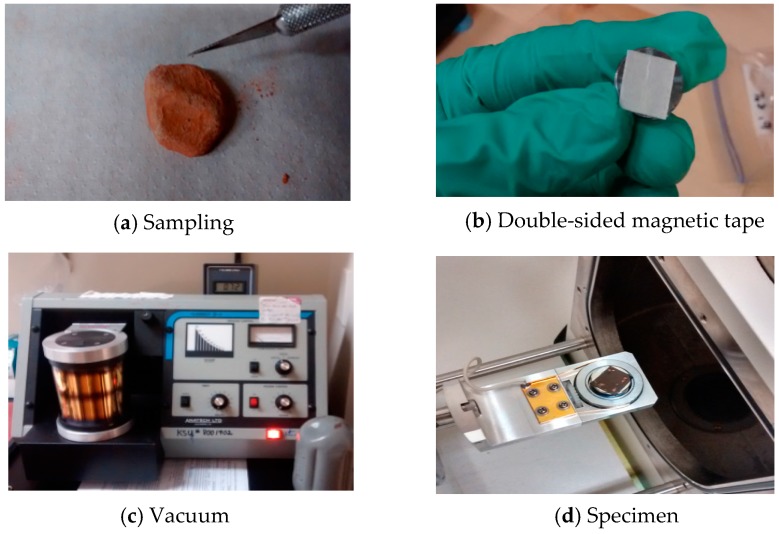
Specimen prepared for scanning electronic microscope (SEM).

To understand the physical properties of the aggregates sintered from dredged material at different temperatures, the microstructures of these aggregates were observed using a Hitachi S-2600N scanning electron microscope (1–30 kV, Tokyo, Japan) hosted at the Liquid Crystal Institute at Kent State University. To prepare for a sample for SEM observation, a small particle was sliced from an aggregate using an X-acto blade (Figure 6a). Then particle was placed on a magnetic double sided tape which was attached to the stage of a vacuum chamber (Figure 6b). The non-conductive surface of the particle required gold coating in the chamber, which was vacuumed to 50 mTorr and purged with argon gas (Figure 6c). The gold atoms were diffused onto the specimen surface with a DC voltage of 6 V applied on the top and bottom of the electrode in pulses. Finally, the specimen was placed into the SEM chamber (Figure 6d). Images of samples were obtained at magnification of 2500×.

### 2.7. Green Roof Material

**Figure 7:**
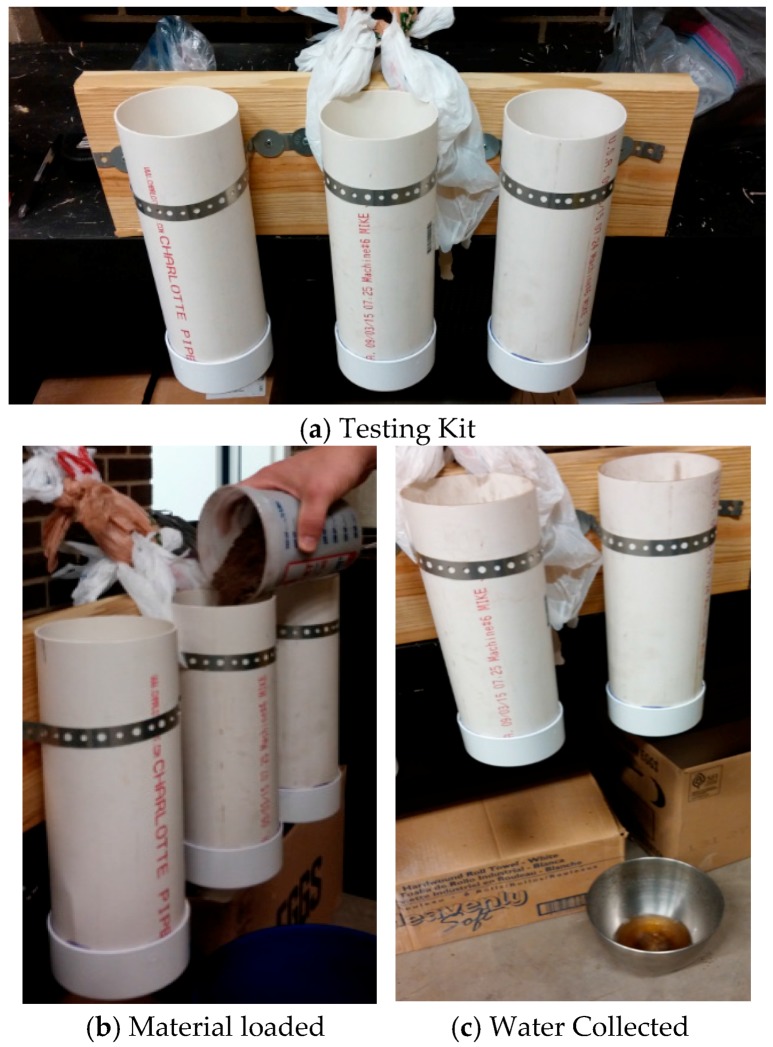
Lab testing for dredge green roof material.

**Figure 8:**
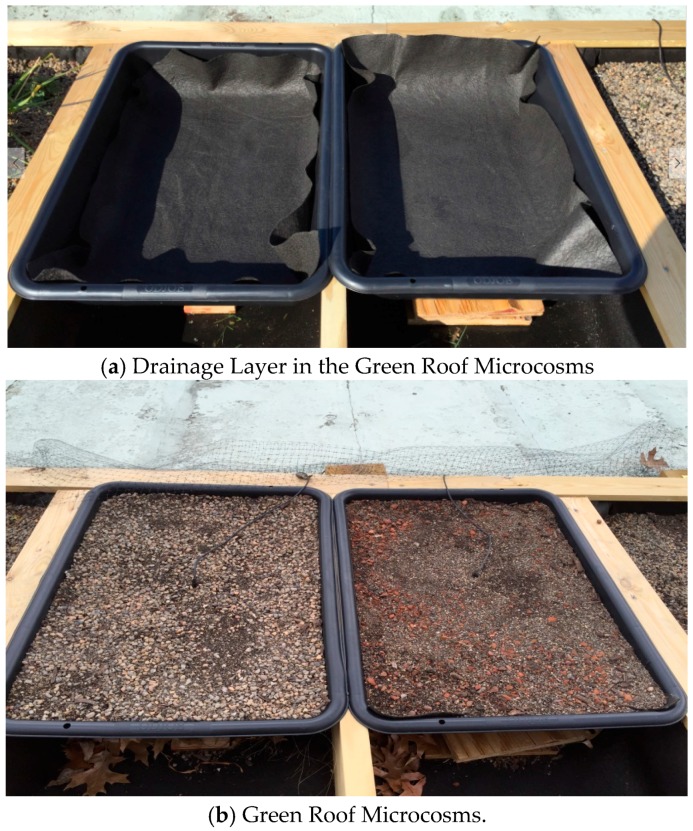
Filed testing of green roof materials.

The LWA made from dredged material was crushed into small sized aggregates and used to create an experimental material by replacing the lightweight mineral aggregate by volume in a conventional green roof material product, Rooflite^®^. Rooflite^®^ uses a balanced blend of LWA (like HydRocks or pumice) and premium organic components. The experimental green roof material made from sintered dredge was tested in the lab for its unit weight, water retention capacity, and leachate water quality. The lab testing device was built with three polyvinyl chloride (PVC) pipes (305 mm (12 inches) long and 100 mm (4 inches) inner diameter) attached to a 25 mm (1 inch) thick wood board (Figure 7a). A wire mesh and a cap with holes were put at the bottom of the PVC pipe, which kept the solids from clogging the holes and facilitated the water drainage. The pipes were filled to a depth of 152 mm (6 inches) with green roof materials, with LWA made from the dredged material (Figure 7b). Distilled water was poured from the top of the pipe. Drained water was collected using a stainless steel bowl on the floor (Figure 7c). Test strips were used to indicate the level of total hardness, total alkalinity, total chlorine, and the pH level. A test to gauge nitrogen levels in the form of nitrate and nitrite was also performed. In addition, two green roof microcosms were installed for field testing at the Cleveland Industrial Innovation Center using the experimental green roof material and Rooflite^®^. The soil moisture contents in the two microcosms were measured using moisture probes and recorded by a data logger for six weeks. Two plant growing tubs with depths 150 mm (6 inches) each were prepared for the Rooflite^®^ material (left) and dredge green roof material (right) with a separate drainage course topped with a synthetic drainage layer (Figure 8a). Small holes were drilled at the lower left corner to drain the excess water off the tub. The two tubs were filled with materials to the top and watered to the saturation on the first day of installation. Two moisture sensors were inserted at the center of the tubs to record the moisture levels in the two tubs for six weeks (Figure 8b). 

## 3. Experimental Results and Discussion

### 3.1. Heavy Metals

The sampling location for S1, S2, and S3 in the CDF is indicated as the red triangle in Figure 1. The contents of heavy metals from the three sampling depths are listed and compared with the Risk Screen Levels (RSL) specified by U.S. Environmental Protection Agency (USEPA) for industrial and residential uses in Table 1. The RSLs are presented with target cancer risk (TR) of 1 × 10^−6^ and with target hazard quotient (THQ) of 0.1. The contents of the majority of heavy metals are lower than the RSL specified for residential uses except arsenic, iron, and manganese. The levels of arsenic from the three samples are higher than the industrial RSL. The RSLs for heavy metals specified by USEPA are more stringent than soil direct contact standards for industrial and residential uses specified in Ohio EPA’s Voluntary Action Programs (VAP). For example, the upper limits for arsenic listed in 2014 Ohio VAP are 77 mg/kg for the industrial direct contact, and 12 mg/kg for the residential direct contact. The tested arsenic levels are below the Ohio VAP value for industrial. The aggregates made from dredged material are expected to be used in the green infrastructure construction to manage stormwater in post-industrial brownfields. In addition, the sintering process to manufacture the aggregate is believed to immobilize the heavy metals in the crystalline structure of the aggregate. The leaching test performed by Wei [18] implies that the residual amounts of heavy metals are difficult to leach from the sintered LWA samples, because the heavy metals were efficiently solidified within the silicate or aluminosilicate matrix, which was also confirmed by X-ray powder diffraction testing. Therefore, the toxicity risk of the aggregates sintered from the dredged material due to the heavy metals is low if used in the urban brownfields. 

Table 1 also indicates that the levels of aluminum, calcium, iron, and magnesium are relatively high. Literatures [19,20,21,22,23] state these elements have excellent phosphorus absorption potential. Great Lakes including Lake Erie have been suffering from recurrent harmful algal blooms since the mid-1990s, which is caused by nutrient-rich stormwater runoff. Excess nutrients, particularly phosphorus, are believed to contribute to the algal growth, which is revealed by Heidelberg University’s long- term tributary monitoring program on dissolved reactive phosphorus in the Great Lakes. Green infrastructure constructed using the aggregate which is made from the dredged material may also have potential to improve the water quality by removing phosphorus from the stormwater runoff. 

### 3.2. Grain Size Distribution

Samples were collected from different locations with a depth of 0.9–1.8 m (3–6 ft) in the CDF for the sieve analysis. The results of sieve analyses are summarized in Table 2. At the sampling site of S1, S2, and S3, the grain size distribution indicates the material is very sandy and most are gravel and sand. Table 2 shows the material in the northern and eastern zone of CDF 12 (NE average) is much coarser than that in the central and western area (CW average). Sample S4 was taken from the central and western area in the CDF, which included more than 70% silt and clay. Clay was used as a bonding agent to mix with S1, S2, and S3 to make the aggregate, while S4 did not need clay due to its initial high silt and clay contents. After the dredged material samples were dried and pulverized, a no. 16 sieve (1.19 mm sieve opening size) was used to remove plant roots, gravel and coarse sand.

### 3.3. Thermal Analysis (TGA and DSC)

**Figure 9:**
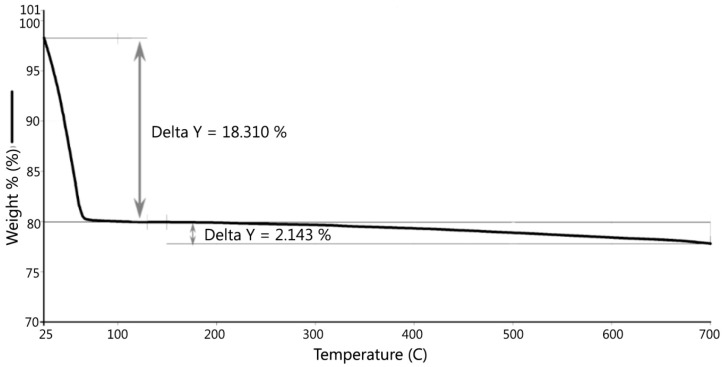
TGA.

**Figure 10:**
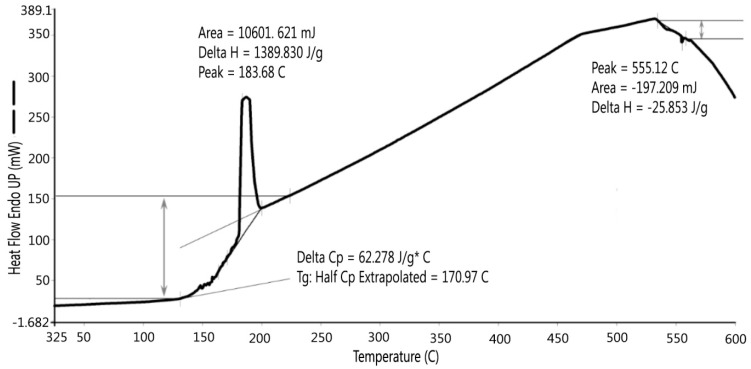
Differential Scanning Calorimetry (DSC).

TGA and DSC were performed for S3 and the results are presented in Figure 9 and Figure 10. Figure 9 shows there was an initial 2% weight loss at 25 °C, probably due to the evaporation of water. The sample weight did not decrease between 100 °C and 200 °C. Between 200 °C and 700 °C, the weight loss was 2.143% due to the loss of crystalline water and burning of organics in the dredged material. DSC testing indicated two peaks, as shown in Figure 10. The first peak occurred at 184 °C, which was caused by the loss of absorbed water stored in micro-holes in the minerals included in the dredged material, and the second peak at 555 °C was possibly due to the loss of crystalline water and organics in the dredged material. Gases were formed due to the water and organic losses when the dredged material was heated to the point of incipient fusion; the gases were trapped in the melted material. At a higher sintering temperature, a LWA with crystalline structure can be produced. Therefore, it is recommended to pre-heat the dredged material at least to 550 °C to generate the gases and heat the material to the point of incipient fusion. 

### 3.4. Specific Gravity

According to the thermal analysis, the aggregates need to be preheated at 550 °C. Then they need to be sintered at a higher temperature to gain sufficient strength. S1, S2, and S3 samples were sandy dredged material. They were mixed with 0%, 5%, 10%, 15%, and 20% clay and sintered at 550 °C, 1000 °C, 1050 °C, 1100 °C, and 1150 °C. It was noticed that all aggregates sintered at 550 °C did not gain enough strength. This is because the strong micro crystalline structure of the aggregate is formed at higher temperatures with minerals fused. The high clay replacements with 15% and 20% made the mixtures too sticky and increased the overall cost of the production, although very hard aggregates were manufactured. It was determined that only 0%, 5%, and 10% clay would be used as the bonding agent in the mixtures with S1, S2, and S3. S4 sample dredge had high silt and clay contents. It was mixed with water to make the small balls. After all fresh balls were air-dried for at least 24 h, they were preheated to 550 °C and then sintered to 1000 °C, 1050 °C, 1100 °C, and 1150 °C. These sample aggregates and selected aggregates made from S1, S2, and S3 were tested for their physical properties.

The specific gravities (SGs) and water absorption rates of selected samples were tested; results are summarized in Table 3. In Table 3, S1_5%_1100 means 5% of S1 by weight was replaced by clay, and the aggregate was sintered at 1100 °C. The SGs of all aggregate samples range between 1.46 and 1.74, and water absorption rates fall in the range between 10.96% and 23.40%. Compared to limestone with SG of 2.6–3.0, goethite with SG of 3.4–4.0, and limonite with SG of 4.0–4.8, the aggregates made from dredged material are lightweight. The dredged material can be sintered over a relatively wide range of temperatures to produce LWA.

**Figure 11:**
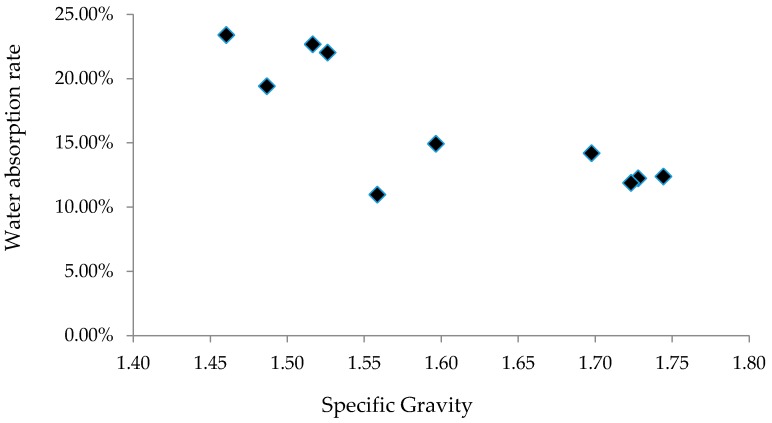
Water absorption vs. specific gravity.

The relationship between SG and water absorption rate of the LWA made from dredged material is illustrated in Figure 11. As the SG increases, water absorption rate decreases. When SG of aggregates increases, the size of micro-holes decreases, thus the water absorption rate is reduced. Table 3 also shows that as the clay content increases (S2_0%_1100, S2_5%_1100, and S2_10%_1100), the SG of the LWA increases, and the water absorption rate decreases. Aggregates made from S4 were sintered at temperatures varying from 1000 °C to 1150 °C. When it was sintered at 1100 °C, its SG reached the maximum value but its water absorption rate was at the minimum value. The maximum SG was expected to occur when the aggregates were sintered at about 1100 °C. The change in nature of the porosity is expected to occur at about 1100 °C, which leads to reduced SG at the temperature of 1150 °C. 

### 3.5. SEM

**Figure 12:**
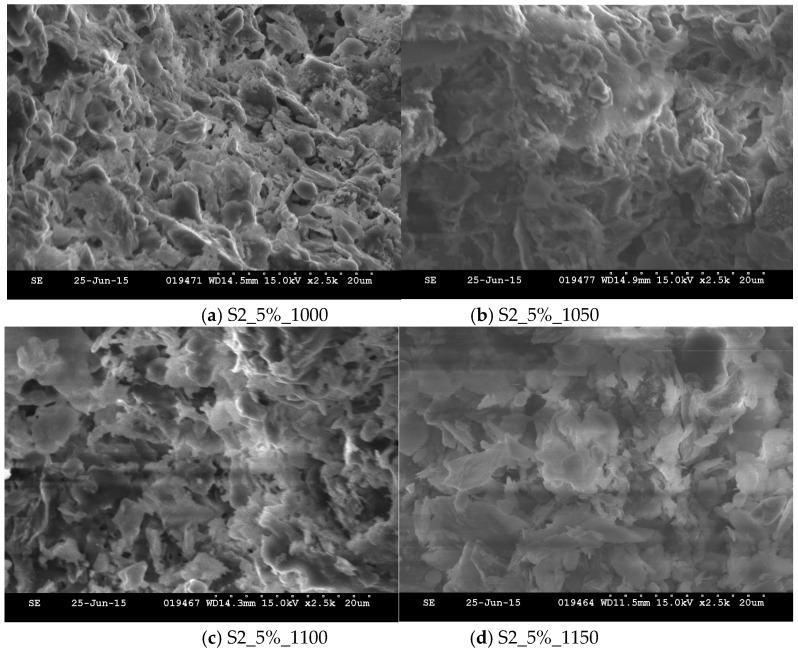
SEM S2_5% with varying sintering temperatures with magnification of 2500×.

Figure 12 shows SEM images, at a magnification of 2500×, of aggregates made of S2_5% sintered at different temperatures. As the temperature increases, the nature of porosity in the aggregate changes. When the temperature increased from 1000 °C to 1050 °C, the pores were formed on the surface of the aggregate and they were connected. When the temperature increased from 1050 °C to 1100 °C, the pores were discontinued and isolated by enamels with higher density. The isolated pores were enlarged when the temperature was raised to 1150 °C. This may explain why the SG of aggregates reached the maximum value at about 1100 °C. After the peak, the SG reduced due to the isolated enlarged pores. 

### 3.6. Green Roof Material

The lab testing has proved that LWA can be produced using dredged material sampled from the CDF in Cleveland. Depending on the grain size distribution, clay may or may not be used in the mixtures as a bonding agent. S4, with high silt and clay contents, was used for LWA mass production. Although lightweight aggregates can be manufactured using a relatively wide temperature range, 1150 °C was selected because of the low SG and relatively high water absorption rate. The LWA in Rooflite^®^ was replaced by the LWA made from the S4 dredge sample. Lab testing and field testing were performed to evaluate the performance of the green roof growing media made from the dredged material.

The unit weight, dead load due to the material, and water retention capacity were examined using the testing kit shown in Figure 7. The lab testing results are summarized in Table 4. The water retention capacity is about 25.50% by weight. The unit weight of Rooflite^®^ ranges between 44 lb/ft^3^ (705 kg/m^3^) and 53 lb/ft^3^ (849 kg/m^3^) [24]. The green roof material made from dredged material is heavier than Rooflite^®^ due to higher SG of the LWA made from dredged material. 

The pH, total alkalinity, total chlorine, total hardness, and nitrogen from nitrate and nitrite of the leachate water from the columns were measured using test strips and compared with distilled water. The pH, levels of total alkalinity, total chlorine, and nitrogen from nitrite are similar to distilled water. But the hardness, and total of nitrate nitrogen and nitrite nitrogen were much higher. Hardness ranged between 250 mg/L and 425 mg/L while distilled water was 0 mg/L, and the total of nitrate nitrogen and nitrite nitrogen was >50 mg/L compared to 0 mg/L in the distilled water. 

**Figure 13:**
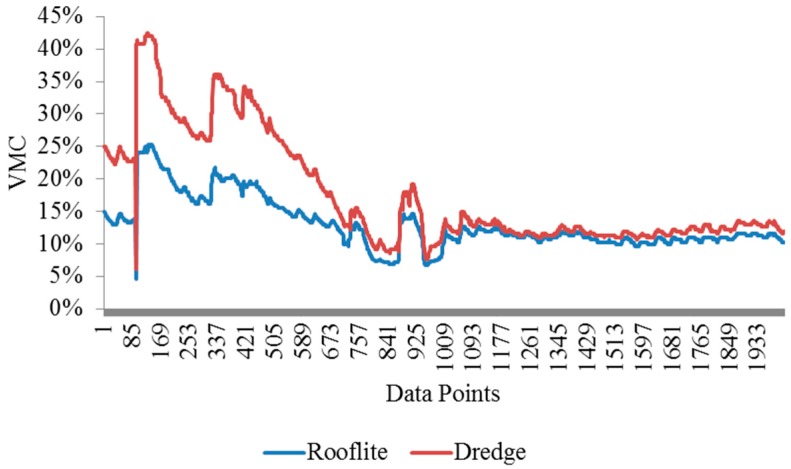
Soil volumetric moisture content (%) rooflite vs. the experimental.

The soil volumetric moisture contents (VMC%) in the Rooflite^®^ and experimental green roof material were measured using moisture sensors. The two tubes were monitored for six weeks and the readings were plotted and shown in Figure 13. The experimental green roof material exhibits a consistently higher soil VMC than the Rooflite^®^. The first two peaks were from controlled irrigation events and the third peak was due to a rain event. The field implement shows the dredge green roof material has comparable performance with Rooflite^®^ in terms of water retention.

## 4. Conclusions and Recommendations

The reuse potential of dredged material from Cuyahoga River and Harbor of Cleveland in green roof construction to manage stormwater runoff was evaluated in this project. A comprehensive experimental plan was proposed by the research team to examine the suitability of the raw dredge in LWA manufacturing, and the performance of the LWA in green roof. 

The chemical analyses on heavy metals confirmed there is a low risk to reuse the dredged material in the green infrastructure installed in post-industrial brownfield. The grain size distributions of the dredged material may indicate different potential beneficial uses. For LWA manufacturing, the sandy dredged material can be mixed with certain amount of clay, while clay is not needed for the dredge with high silt and clay contents. The thermal analysis performed for the dredge samples in this study indicated that the fresh aggregates should be preheated at 550 °C to convert crystal water and organics to gases at the point of incipient fusion of the minerals in the dredged material. A higher sintering temperature ranging from 1000 °C to 1150 °C is required to produce a LWA with sufficient strength. The temperature of 1150 °C was selected in this study as an optimum sintering temperature because it generated a LWA with low SG and high water absorption rate. Water absorption rate decreases as the SG increases. The sintering temperature changes the nature of the porosity of the LWA as revealed in the SEM imaging. The dredge green roof material has a comparable hydrologic performance with the Rooflite^®^, a conventional growing media for green roof construction. This project shows that dredged material has great potential to be used for LWA manufacturing, which can be used for green infrastructure construction. 

The chemical analyses showed relatively high levels of iron, aluminum, calcium, and magnesium contents in the dredged material sampling from the CDF 12 in Cleveland. Those elements are reported to have phosphorus absorption potential. The LWA made from the dredged material may have a potential to remove the phosphorus out of the stormwater runoff to improve the stormwater quality. Mineral admixtures may be incorporated in and mixed with the raw dredge to produce a LWA with nutrients removal capability. The current study focused on the function of stormwater management of sintered material in a green roof growing course. Vegetation should be further examined in field trials to identify species suitable for this growing media. Additionally, plant studies using raw dredge from Cuyahoga River and/or Harbor of Cleveland would provide cities, agencies, and nursery trades with information needed to determine proper co-benefit targets. An economic and life cycle cost-and-benefit analysis would determine the investment opportunities and constraints for a Lake Erie or Great Lakes market product of a sintered dredge product. The LWA sintered from the dredged material possesses promising traits of hydrological and structural capacities which demand further investigation into various forms of bioretention mixes used in green infrastructure. It is possible new types of green infrastructure and stormwater control measures could be developed using the LWA sintered from the dredged material. 

## Figures and Tables

**Table 1:** Heavy metal contents.

Heavy Metals	Soil RSL Industrial	Residential	0’–3’	3’–6’	6’–9’
	TR = 1 × 10^−6^, THQ = 0.1 (mg/kg)		(mg/kg)	(mg/kg)	(mg/kg)
Aluminum	110,000	7700	3700	2700	6700
Antimony	47	3.1	<0.43	<0.44	<0.52
Arsenic	3	0.67	7.4	6.4	11
Barium	22,000	1500	24	21	53
Beryllium	230	16	0.34	0.34	0.43
Cadmium	98	7	0.85	0.31	1
Calcium	NS	NS	5300	7100	11,000
Chromium	NS	NS	11	22	18
Cobalt	35	2.3	4.9	3.8	8.3
Copper	4700	310	42	49	36
Iron	82,000	5500	15,000	16,000	22,000
Lead	800	400	14	15	31
Magnesium	NS	NS	2000	2400	4200
Manganese	2600	180	210	380	460
Nickel Soluble Salts	2200	150	28	18	27
Potassium	NS	NS	430	310	960
Selenium	580	39	0.59	<0.31	0.54
Silver	580	39	<0.056	<0.058	0.078
Sodium	NS	NS	79	160	120
Thalium	1.2	0.078	0.21	0.13	0.18
Vanadium	580	39	8.9	13	14
Zinc	35,000	2300	100	87	170
Mercury	4	0.94	0.026	0.021	0.093
Total Cyanide	13	2.1	0.46	<0.31	<0.44
Chromium(VI)	6.3	0.3	<0.31	0.41	<0.37

**Table 2:** Grain size distribution.

Samples	Gravel	Coarse Sand	Fine Sand	Silt	Clay	Total
	(3” to #10)	#10 to #40	#40 to #200	#200 to 0.005	>0.005 mu	
	%	%	%	%	%	%
3’–6’	41.7	23.5	29.3	4.0	1.4	99.9
NE Average	14.4	16.2	49.9	13.5	6.1	100.0
CW Average	0.1	2.2	23.6	48.3	25.9	100.0

**Table 3:** Specific gravity and water absorption rate.

Samples	Temperature (°C)	Specific Gravity	Water Absorption (%)
S1_5%_1100	1100	1.70	14.20
S1_10%_1100	1100	1.74	12.37
S2_0%_1100	1100	1.46	23.40
S2_5%_1100	1100	1.73	12.24
S2_10%_1100	1100	1.72	11.88
S3_10%_1100	1100	1.60	14.93
S4_0%_1000	1000	1.52	22.68
S4_0%_1050	1050	1.53	22.02
S4_0%_1100	1100	1.56	10.96
S4_0%_1150	1150	1.49	19.41

**Table 4:** Green roof material made from dredged material.

Specimens	Material Weight	Water Adsorbed	Unit Weight		Dead Load		Dead Load (Saturated)		Water Retention
	(lb)	(mL)	(lb/ft^3^)	(kg/m^3^)	(lb/ft^2^)	(kg/m^2^)	(lb/ft^2^)	(kg/m^2^)	(%)
Column 1	2.29	270	52.40	839.30	26.20	127.91	33.02	161.21	26.04%
Column 2	2.39	280	54.72	876.53	27.36	133.58	34.43	168.12	25.85%
Column 3	2.42	270	55.43	887.86	27.71	135.31	34.53	168.61	24.61%
Average	2.36	273	54.18	867.89	27.09	132.27	34.00	165.98	25.50%
Std Dev.	0.06	4.71	1.29	20.74	0.65	3.16	0.69	3.38	0.63%
